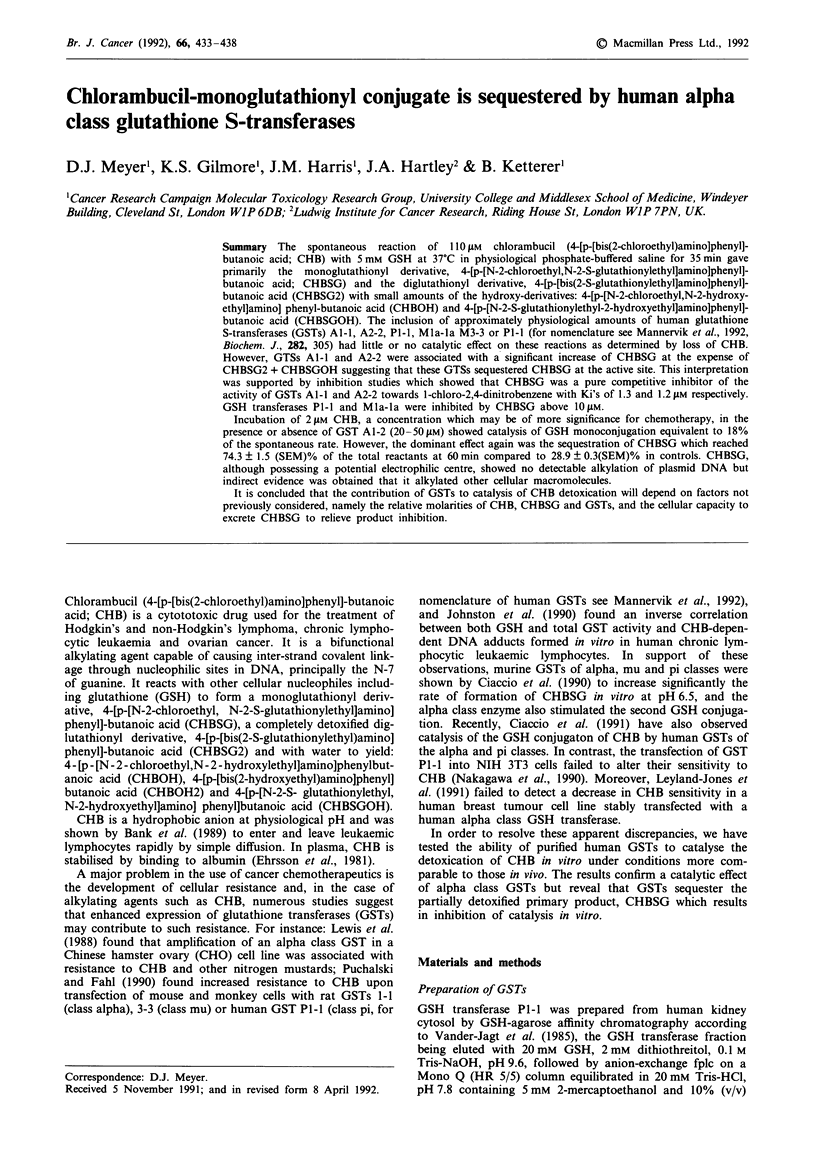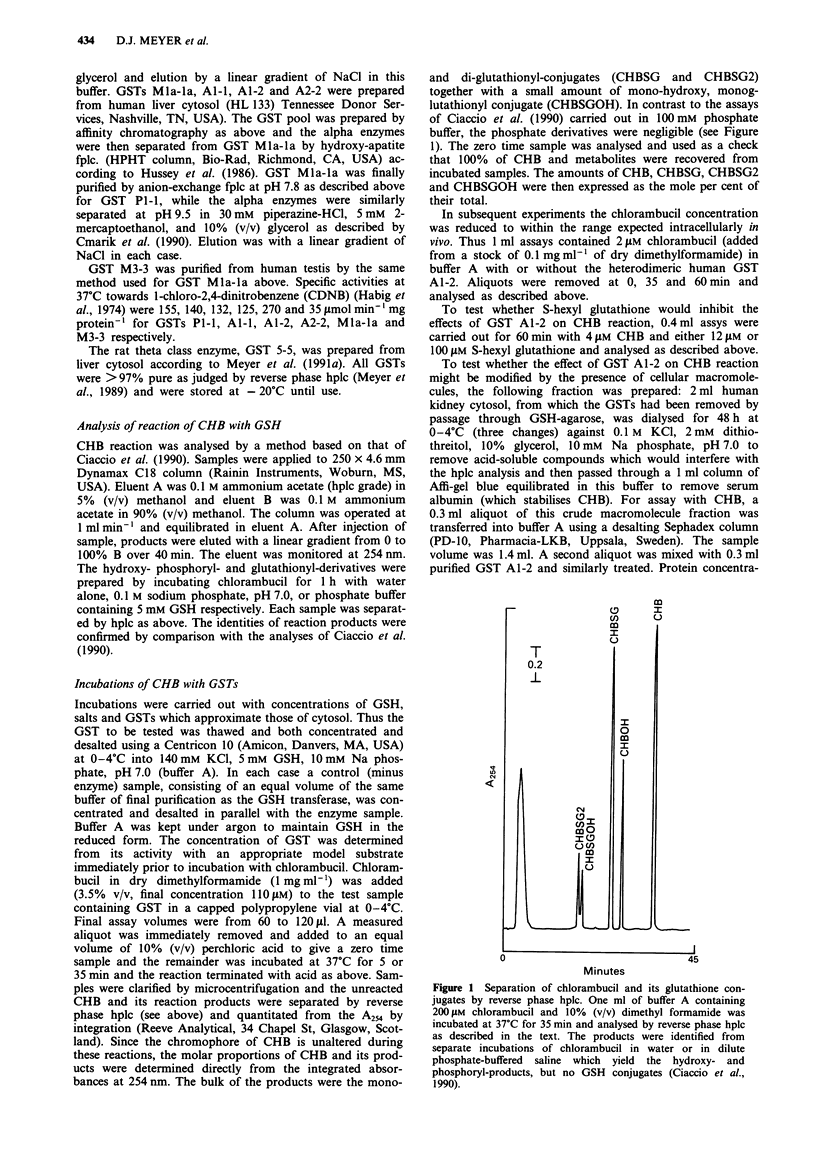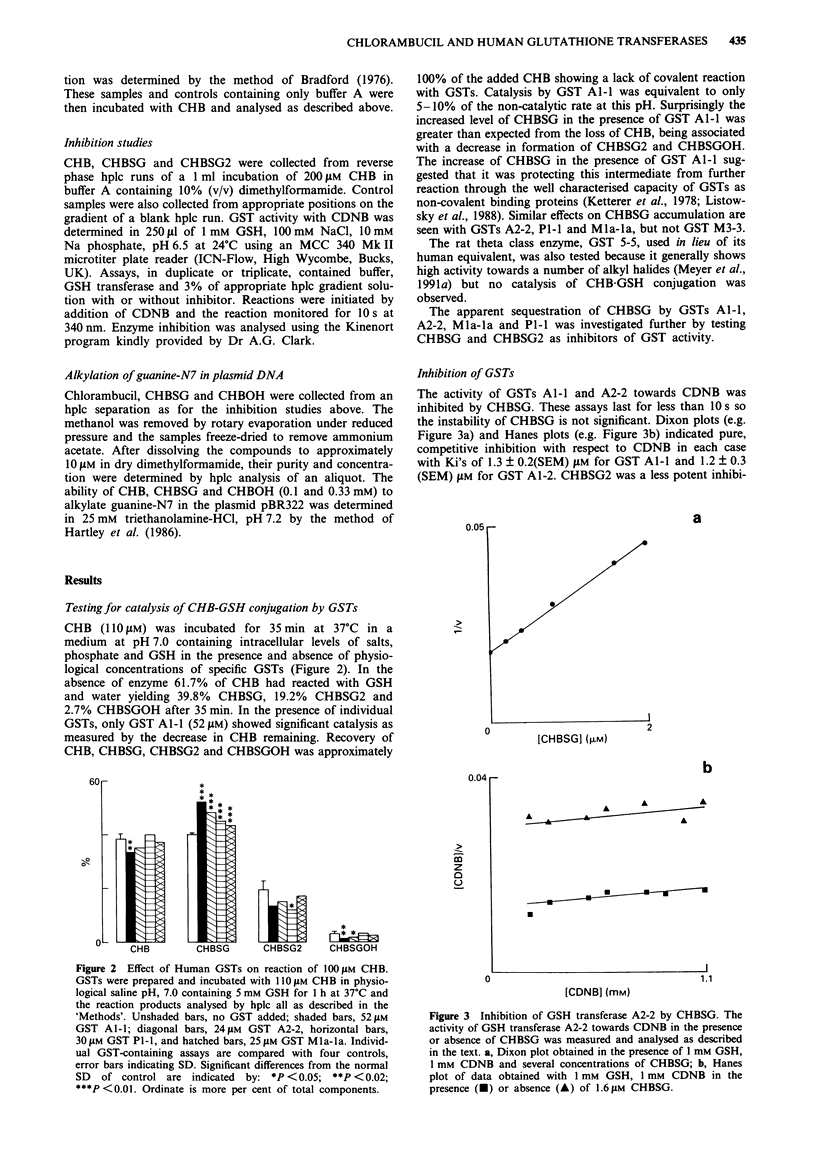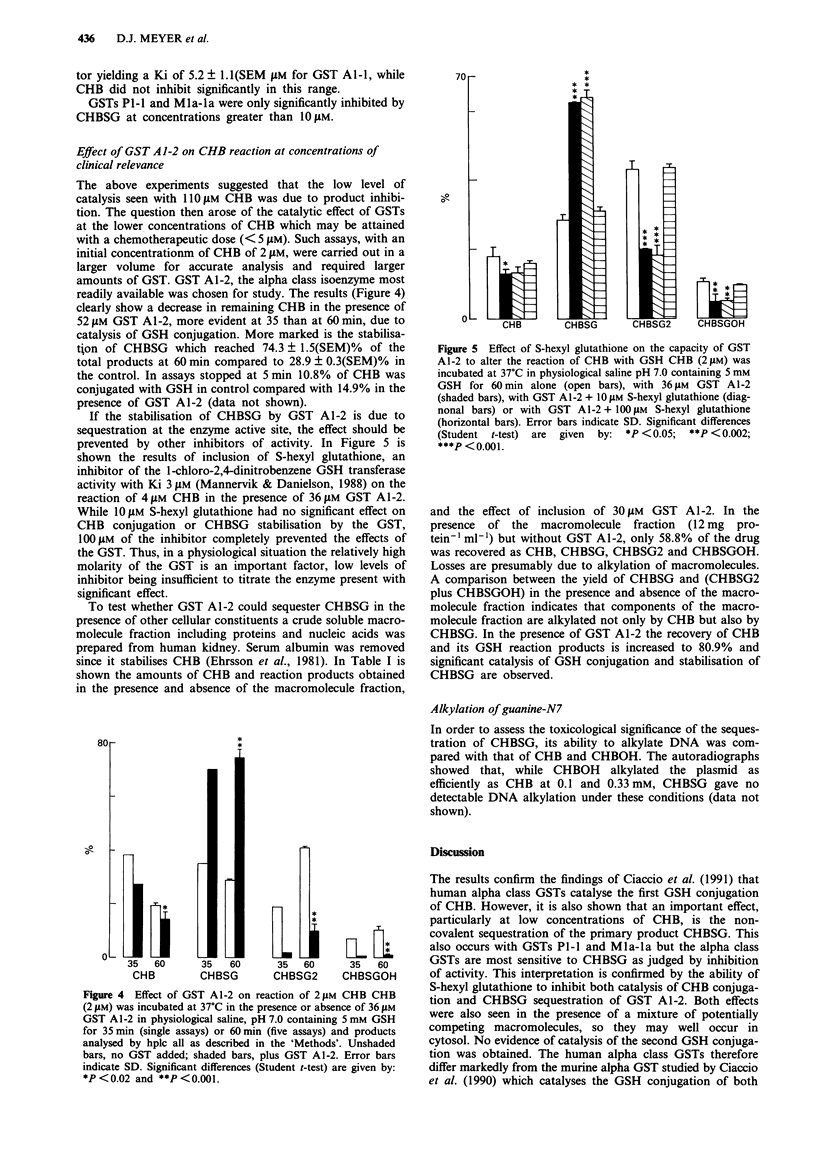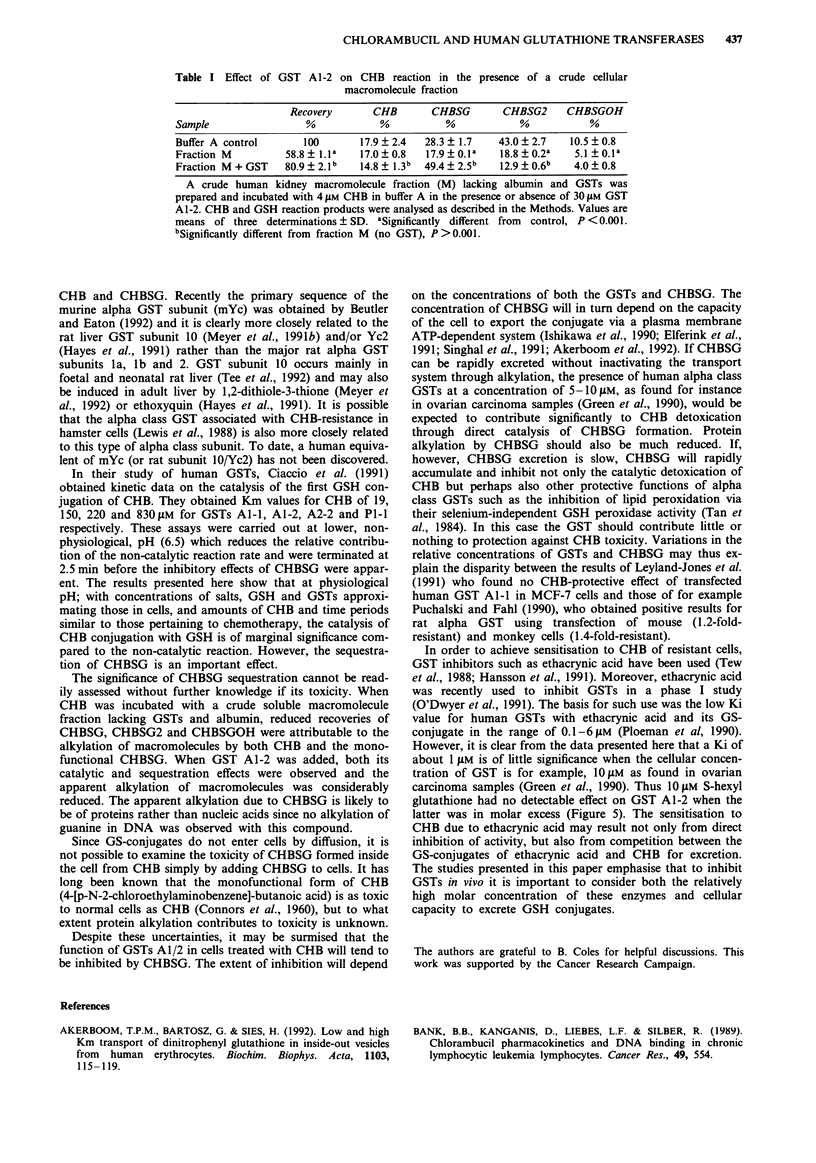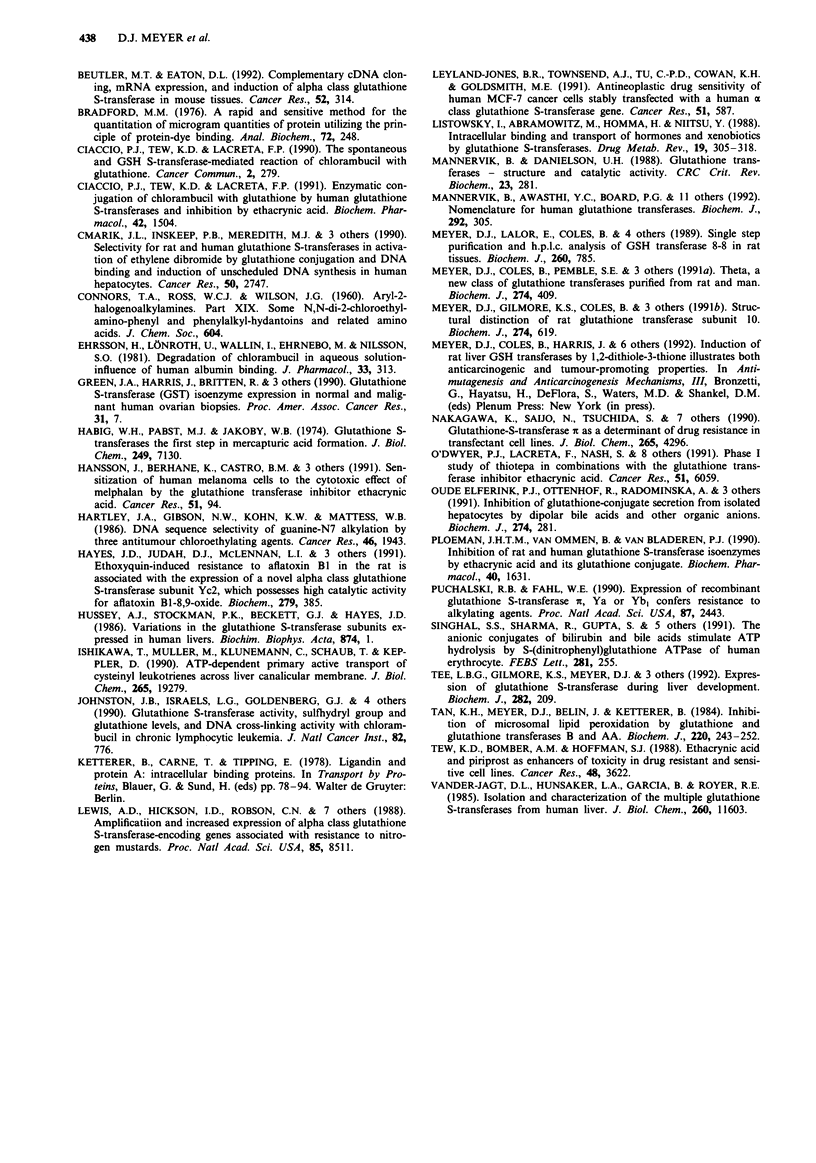# Chlorambucil-monoglutathionyl conjugate is sequestered by human alpha class glutathione S-transferases.

**DOI:** 10.1038/bjc.1992.292

**Published:** 1992-09

**Authors:** D. J. Meyer, K. S. Gilmore, J. M. Harris, J. A. Hartley, B. Ketterer

**Affiliations:** Cancer Research Campaign Molecular Toxicology Research Group, University College and Middlesex School of Medicine, London, UK.

## Abstract

The spontaneous reaction of 110 microM chlorambucil (4-[p-[bis(2-chloroethyl)amino]phenyl]-butanoic acid; CHB) with 5 mM GSH at 37 degrees C in physiological phosphate-buffered saline for 35 min gave primarily the monoglutathionyl derivative, 4-[p-[N-2-chloroethyl,N-2-S-glutathionylethyl]amino]phenyl]-butano ic acid; CHBSG) and the diglutathionyl derivative, 4-[p-[bis(2-S-glutathionylethyl]amino]phenyl]-butanoic acid (CHBSG2) with small amounts of the hydroxy-derivatives: 4-[p-[N-2-chloroethyl,N-2-hydroxy-ethyl]amino] phenyl-butanoic acid (CHBOH) and 4-[p-[N-2-S-glutathionylethyl-2-hydroxyethyl]amino]phenyl]-butanoi c acid (CHBSGOH). The inclusion of approximately physiological amounts of human glutathione S-transferases (GSTs) A1-1, A2-2, P1-1, M1a-1a M3-3 or P1-1 (for nomenclature see Mannervik et al., 1992, Biochem. J., 282, 305) had little or no catalytic effect on these reactions as determined by loss of CHB. However, GTSs A1-1 and A2-2 were associated with a significant increase of CHBSG at the expense of CHBSG2 + CHBSGOH suggesting that these GTs sequestered CHBSG at the active site. This interpretation was supported by inhibition studies which showed that CHBSG was a pure competitive inhibitor of the activity of GSTs A1-1 and A2-2 towards 1-chloro-2,4-dinitrobenzene with Ki's of 1.3 and 1.2 microM respectively. GSH transferases P1-1 and M1a-1a were inhibited by CHBSG above 10 microM. Incubation of 2 microM CHB, a concentration which may be of more significance for chemotherapy, in the presence or absence of GST A1-2 (20-50 microM) showed catalysis of GSH monoconjugation equivalent to 18% of the spontaneous rate. However, the dominant effect again was the sequestration of CHBSG which reached 74.3 +/- 1.5 (SEM)% of the total reactants at 60 min compared to 28.9 +/- 0.3(SEM)% in controls. CHBSG, although possessing a potential electrophilic centre, showed no detectable alkylation of plasmid DNA but indirect evidence was obtained that it alkylated other cellular macromolecules. It is concluded that the contribution of GSTs to catalysis of CHB detoxication will depend on factors not previously considered, namely the relative molarities of CHB, CHBSG and GSTs, and the cellular capacity to excrete CHBSG to relieve product inhibition.


*Br. J. Cancer* (1992), **66**, 433–438 © Macmillan Press Ltd., 1992

### Chlorambucil-monoglutathionyl conjugate is sequestered by human alpha class glutathione S-transferases

D.J. Meyer[1], K.S. Gilmore[1], J.M. Harris[1], J.A. Hartley[2] & B. Ketterer[1]

[1]*Cancer Research Campaign Molecular Toxicology Research Group, University College and Middlesex School of Medicine, Windeyer Building, Cleveland St, London W1P 6DB;* [2]*Ludwig Institute for Cancer Research, Riding House St, London W1P 7PN, UK.*

**Summary** The spontaneous reaction of 110 μM chlorambucil (4-[p-[bis(2-chloroethyl)amino]phenyl]-butanoic acid; CHB) with 5 mM GSH at 37°C in physiological phosphate-buffered saline for 35 min gave primarily the monoglutathionyl derivative, 4-[p-[N-2-chloroethyl,N-2-S-glutathionylethyl]amino]phenyl]-butanoic acid; CHBSG) and the diglutathionyl derivative, 4-[p-[bis(2-S-glutathionylethyl]amino]phenyl]-butanoic acid (CHBSG2) with small amounts of the hydroxy-derivatives: 4-[p-[N-2-chloroethyl,N-2-hydroxy-ethyl]amino] phenyl-butanoic acid (CHBOH) and 4-[p-[N-2-S-glutathionylethyl-2-hydroxyethyl]amino]phenyl]-butanoic acid (CHBSGOH). The inclusion of approximately physiological amounts of human glutathione S-transferases (GSTs) A1-1, A2-2, P1-1, M1a-1a M3-3 or P1-1 (for nomenclature see Mannervik *et al.*, 1992, *Biochem. J.*, **282**, 305) had little or no catalytic effect on these reactions as determined by loss of CHB. However, GTSs A1-1 and A2-2 were associated with a significant increase of CHBSG at the expense of CHBSG2 + CHBSGOH suggesting that these GTSs sequestered CHBSG at the active site. This interpretation was supported by inhibition studies which showed that CHBSG was a pure competitive inhibitor of the activity of GSTs A1-1 and A2-2 towards 1-chloro-2,4-dinitrobenzene with Ki's of 1.3 and 1.2 μM respectively. GSH transferases P1-1 and M1a-1a were inhibited by CHBSG above 10 μM.

Incubation of 2 μM CHB, a concentration which may be of more significance for chemotherapy, in the presence or absence of GST A1-2 (20–50 μM) showed catalysis of GSH monoconjugation equivalent to 18% of the spontaneous rate. However, the dominant effect again was the sequestration of CHBSG which reached 74.3 ± 1.5 (SEM)% of the total reactants at 60 min compared to 28.9 ± 0.3(SEM)% in controls. CHBSG, although possessing a potential electrophilic centre, showed no detectable alkylation of plasmid DNA but indirect evidence was obtained that it alkylated other cellular macromolecules.

It is concluded that the contribution of GSTs to catalysis of CHB detoxication will depend on factors not previously considered, namely the relative molarities of CHB, CHBSG and GSTs, and the cellular capacity to excrete CHBSG to relieve product inhibition.

Chlorambucil (4-[p-[bis(2-chloroethyl)amino]phenyl]-butanoic acid; CHB) is a cytototoxic drug used for the treatment of Hodgkin's and non-Hodgkin's lymphoma, chronic lymphocytic leukaemia and ovarian cancer. It is a bifunctional alkylating agent capable of causing inter-strand covalent linkage through nucleophilic sites in DNA, principally the N-7 of guanine. It reacts with other cellular nucleophiles including glutathione (GSH) to form a monoglutathionyl derivative, 4-[p-[N-2-chloroethyl, N-2-S-glutathionylethyl]amino] phenyl]-butanoic acid (CHBSG), a completely detoxified diglutathionyl derivative, 4-[p-[bis(2-S-glutathionylethyl)amino] phenyl]-butanoic acid (CHBSG2) and with water to yield: 4-[p-[N-2-chloroethyl,N-2-hydroxylethyl]amino]phenylbutanoic acid (CHBOH), 4-[p-[bis(2-hydroxyethyl)amino]phenyl] butanoic acid (CHBOH2) and 4-[p-[N-2-S- glutathionylethyl, N-2-hydroxyethyl]amino] phenyl]butanoic acid (CHBSGOH).

CHB is a hydrophobic anion at physiological pH and was shown by Bank *et al.* (1989) to enter and leave leukaemic lymphocytes rapidly by simple diffusion. In plasma, CHB is stabilised by binding to albumin (Ehrsson *et al.*, 1981).

A major problem in the use of cancer chemotherapeutics is the development of cellular resistance and, in the case of alkylating agents such as CHB, numerous studies suggest that enhanced expression of glutathione transferases (GSTs) may contribute to such resistance. For instance: Lewis *et al.* (1988) found that amplification of an alpha class GST in a Chinese hamster ovary (CHO) cell line was associated with resistance to CHB and other nitrogen mustards; Puchalski and Fahl (1990) found increased resistance to CHB upon transfection of mouse and monkey cells with rat GSTs 1-1 (class alpha), 3-3 (class mu) or human GST P1-1 (class pi, for nomenclature of human GSTs see Mannervik *et al.*, 1992), and Johnston *et al.* (1990) found an inverse correlation between both GSH and total GST activity and CHB-dependent DNA adducts formed *in vitro* in human chronic lymphocytic leukaemic lymphocytes. In support of these observations, murine GSTs of alpha, mu and pi classes were shown by Ciaccio *et al.* (1990) to increase significantly the rate of formation of CHBSG *in vitro* at pH 6.5, and the alpha class enzyme also stimulated the second GSH conjugation. Recently, Ciaccio *et al.* (1991) have also observed catalysis of the GSH conjugaton of CHB by human GSTs of the alpha and pi classes. In contrast, the transfection of GST P1-1 into NIH 3T3 cells failed to alter their sensitivity to CHB (Nakagawa *et al.*, 1990). Moreover, Leyland-Jones *et al.* (1991) failed to detect a decrease in CHB sensitivity in a human breast tumour cell line stably transfected with a human alpha class GSH transferase.

In order to resolve these apparent discrepancies, we have tested the ability of purified human GSTs to catalyse the detoxication of CHB *in vitro* under conditions more comparable to those *in vivo*. The results confirm a catalytic effect of alpha class GSTs but reveal that GSTs sequester the partially detoxified primary product, CHBSG which results in inhibition of catalysis *in vitro*.

### Materials and methods

### *Preparation of GSTs*

GSH transferase P1-1 was prepared from human kidney cytosol by GSH-agarose affinity chromatography according to Vander-Jagt *et al.* (1985), the GSH transferase fraction being eluted with 20 mM GSH, 2 mM dithiothreitol, 0.1 M Tris-NaOH, pH 9.6, followed by anion-exchange fplc on a Mono Q (HR 5/5) column equilibrated in 20 mM Tris-HCl, pH 7.8 containing 5 mM 2-mercaptoethanol and 10% (v/v)

Correspondence: D.J. Meyer.
Received 5 November 1991; and in revised form 8 April 1992.

glycerol and elution by a linear gradient of NaCl in this buffer. GSTs M1a-1a, A1-1, A1-2 and A2-2 were prepared from human liver cytosol (HL 133) Tennessee Donor Services, Nashville, TN, USA). The GST pool was prepared by affinity chromatography as above and the alpha enzymes were then separated from GST M1a-1a by hydroxy-apatite fplc. (HPHT column, Bio-Rad, Richmond, CA, USA) according to Hussey *et al.* (1986). GST M1a-1a was finally purified by anion-exchange fplc at pH 7.8 as described above for GST P1-1, while the alpha enzymes were similarly separated at pH 9.5 in 30 mM piperazine-HCl, 5 mM 2-mercaptoethanol, and 10% (v/v) glycerol as described by Cmarik *et al.* (1990). Elution was with a linear gradient of NaCl in each case.

GST M3-3 was purified from human testis by the same method used for GST M1a-1a above. Specific activities at 37°C towards 1-chloro-2,4-dinitrobenzene (CDNB) (Habig *et al.*, 1974) were 155, 140, 132, 125, 270 and 35 $\mu$mol min$^{-1}$ mg protein$^{-1}$ for GSTs P1-1, A1-1, A1-2, A2-2, M1a-1a and M3-3 respectively.

The rat theta class enzyme, GST 5-5, was prepared from liver cytosol according to Meyer *et al.* (1991*a*). All GSTs were >97% pure as judged by reverse phase hplc (Meyer *et al.*, 1989) and were stored at −20°C until use.

### *Analysis of reaction of CHB with GSH*

CHB reaction was analysed by a method based on that of Ciaccio *et al.* (1990). Samples were applied to 250 × 4.6 mm Dynamax C18 column (Rainin Instruments, Woburn, MS, USA). Eluent A was 0.1 M ammonium acetate (hplc grade) in 5% (v/v) methanol and eluent B was 0.1 M ammonium acetate in 90% (v/v) methanol. The column was operated at 1 ml min$^{-1}$ and equilibrated in eluent A. After injection of sample, products were eluted with a linear gradient from 0 to 100% B over 40 min. The eluent was monitored at 254 nm. The hydroxy- phosphoryl- and glutathionyl-derivatives were prepared by incubating chlorambucil for 1 h with water alone, 0.1 M sodium phosphate, pH 7.0, or phosphate buffer containing 5 mM GSH respectively. Each sample was separated by hplc as above. The identities of reaction products were confirmed by comparison with the analyses of Ciaccio *et al.* (1990).

### *Incubations of CHB with GSTs*

Incubations were carried out with concentrations of GSH, salts and GSTs which approximate those of cytosol. Thus the GST to be tested was thawed and both concentrated and desalted using a Centricon 10 (Amicon, Danvers, MA, USA) at 0–4°C into 140 mM KCl, 5 mM GSH, 10 mM Na phosphate, pH 7.0 (buffer A). In each case a control (minus enzyme) sample, consisting of an equal volume of the same buffer of final purification as the GSH transferase, was concentrated and desalted in parallel with the enzyme sample. Buffer A was kept under argon to maintain GSH in the reduced form. The concentration of GST was determined from its activity with an appropriate model substrate immediately prior to incubation with chlorambucil. Chlorambucil in dry dimethylformamide (1 mg ml$^{-1}$) was added (3.5% v/v, final concentration 110 $\mu$M) to the test sample containing GST in a capped polypropylene vial at 0–4°C. Final assay volumes were from 60 to 120 $\mu$l. A measured aliquot was immediately removed and added to an equal volume of 10% (v/v) perchloric acid to give a zero time sample and the remainder was incubated at 37°C for 5 or 35 min and the reaction terminated with acid as above. Samples were clarified by microcentrifugation and the unreacted CHB and its reaction products were separated by reverse phase hplc (see above) and quantitated from the $A_{254}$ by integration (Reeve Analytical, 34 Chapel St, Glasgow, Scotland). Since the chromophore of CHB is unaltered during these reactions, the molar proportions of CHB and its products were determined directly from the integrated absorbances at 254 nm. The bulk of the products were the mono- and di-glutathionyl-conjugates (CHBSG and CHBSG2) together with a small amount of mono-hydroxy, monoglutathionyl conjugate (CHBSGOH). In contrast to the assays of Ciaccio *et al.* (1990) carried out in 100 mM phosphate buffer, the phosphate derivatives were negligible (see Figure 1). The zero time sample was analysed and used as a check that 100% of CHB and metabolites were recovered from incubated samples. The amounts of CHB, CHBSG, CHBSG2 and CHBSGOH were then expressed as the mole per cent of their total.

In subsequent experiments the chlorambucil concentration was reduced to within the range expected intracellularly *in vivo*. Thus 1 ml assays contained 2 $\mu$M chlorambucil (added from a stock of 0.1 mg ml$^{-1}$ of dry dimethylformamide) in buffer A with or without the heterodimeric human GST A1-2. Aliquots were removed at 0, 35 and 60 min and analysed as described above.

To test whether S-hexyl glutathione would inhibit the effects of GST A1-2 on CHB reaction, 0.4 ml assys were carried out for 60 min with 4 $\mu$M CHB and either 12 $\mu$M or 100 $\mu$M S-hexyl glutathione and analysed as described above.

To test whether the effect of GST A1-2 on CHB reaction might be modified by the presence of cellular macromolecules, the following fraction was prepared: 2 ml human kidney cytosol, from which the GSTs had been removed by passage through GSH-agarose, was dialysed for 48 h at 0–4°C (three changes) against 0.1 M KCl, 2 mM dithiothreitol, 10% glycerol, 10 mM Na phosphate, pH 7.0 to remove acid-soluble compounds which would interfere with the hplc analysis and then passed through a 1 ml column of Affi-gel blue equilibrated in this buffer to remove serum albumin (which stabilises CHB). For assay with CHB, a 0.3 ml aliquot of this crude macromolecule fraction was transferred into buffer A using a desalting Sephadex column (PD-10, Pharmacia-LKB, Uppsala, Sweden). The sample volume was 1.4 ml. A second aliquot was mixed with 0.3 ml purified GST A1-2 and similarly treated. Protein concentra-

**Figure 1** Separation of chlorambucil and its glutathione conjugates by reverse phase hplc. One ml of buffer A containing 200 $\mu$M chlorambucil and 10% (v/v) dimethyl formamide was incubated at 37°C for 35 min and analysed by reverse phase hplc as described in the text. The products were identified from separate incubations of chlorambucil in water or in dilute phosphate-buffered saline which yield the hydroxy- and phosphoryl-products, but no GSH conjugates (Ciaccio *et al.*, 1990).

tion was determined by the method of Bradford (1976). These samples and controls containing only buffer A were then incubated with CHB and analysed as described above.

*Inhibition studies*

CHB, CHBSG and CHBSG2 were collected from reverse phase hplc runs of a 1 ml incubation of 200 μM CHB in buffer A containing 10% (v/v) dimethylformamide. Control samples were also collected from appropriate positions on the gradient of a blank hplc run. GST activity with CDNB was determined in 250 μl of 1 mM GSH, 100 mM NaCl, 10 mM Na phosphate, pH 6.5 at 24°C using an MCC 340 Mk II microtiter plate reader (ICN-Flow, High Wycombe, Bucks, UK). Assays, in duplicate or triplicate, contained buffer, GSH transferase and 3% of appropriate hplc gradient solution with or without inhibitor. Reactions were initiated by addition of CDNB and the reaction monitored for 10 s at 340 nm. Enzyme inhibition was analysed using the Kinenort program kindly provided by Dr A.G. Clark.

*Alkylation of guanine-N7 in plasmid DNA*

Chlorambucil, CHBSG and CHBOH were collected from an hplc separation as for the inhibition studies above. The methanol was removed by rotary evaporation under reduced pressure and the samples freeze-dried to remove ammonium acetate. After dissolving the compounds to approximately 10 μM in dry dimethylformamide, their purity and concentration were determined by hplc analysis of an aliquot. The ability of CHB, CHBSG and CHBOH (0.1 and 0.33 mM) to alkylate guanine-N7 in the plasmid pBR322 was determined in 25 mM triethanolamine-HCl, pH 7.2 by the method of Hartley *et al.* (1986).

### Results

*Testing for catalysis of CHB-GSH conjugation by GSTs*

CHB (110 μM) was incubated for 35 min at 37°C in a medium at pH 7.0 containing intracellular levels of salts, phosphate and GSH in the presence and absence of physiological concentrations of specific GSTs (Figure 2). In the absence of enzyme 61.7% of CHB had reacted with GSH and water yielding 39.8% CHBSG, 19.2% CHBSG2 and 2.7% CHBSGOH after 35 min. In the presence of individual GSTs, only GST A1-1 (52 μM) showed significant catalysis as measured by the decrease in CHB remaining. Recovery of CHB, CHBSG, CHBSG2 and CHBSGOH was approximately 100% of the added CHB showing a lack of covalent reaction with GSTs. Catalysis by GST A1-1 was equivalent to only 5–10% of the non-catalytic rate at this pH. Surprisingly the increased level of CHBSG in the presence of GST A1-1 was greater than expected from the loss of CHB, being associated with a decrease in formation of CHBSG2 and CHBSGOH. The increase of CHBSG in the presence of GST A1-1 suggested that it was protecting this intermediate from further reaction through the well characterised capacity of GSTs as non-covalent binding proteins (Ketterer *et al.*, 1978; Listowsky *et al.*, 1988). Similar effects on CHBSG accumulation are seen with GSTs A2-2, P1-1 and M1a-1a, but not GST M3-3.

The rat theta class enzyme, GST 5-5, used *in lieu* of its human equivalent, was also tested because it generally shows high activity towards a number of alkyl halides (Meyer *et al.*, 1991*a*) but no catalysis of CHB·GSH conjugation was observed.

The apparent sequestration of CHBSG by GSTs A1-1, A2-2, M1a-1a and P1-1 was investigated further by testing CHBSG and CHBSG2 as inhibitors of GST activity.

*Inhibition of GSTs*

The activity of GSTs A1-1 and A2-2 towards CDNB was inhibited by CHBSG. These assays last for less than 10 s so the instability of CHBSG is not significant. Dixon plots (e.g. Figure 3a) and Hanes plots (e.g. Figure 3b) indicated pure, competitive inhibition with respect to CDNB in each case with Ki's of 1.3 ± 0.2(SEM) μM for GST A1-1 and 1.2 ± 0.3 (SEM) μM for GST A1-2. CHBSG2 was a less potent inhibi-

**Figure 2** Effect of Human GSTs on reaction of 100 μM CHB. GSTs were prepared and incubated with 110 μM CHB in physiological saline pH, 7.0 containing 5 mM GSH for 1 h at 37°C and the reaction products analysed by hplc all as described in the 'Methods'. Unshaded bars, no GST added; shaded bars, 52 μM GST A1-1; diagonal bars, 24 μM GST A2-2, horizontal bars, 30 μM GST P1-1, and hatched bars, 25 μM GST M1a-1a. Individual GST-containing assays are compared with four controls, error bars indicating SD. Significant differences from the normal SD of control are indicated by: *$P<0.05$; **$P<0.02$; ***$P<0.01$. Ordinate is more per cent of total components.

**Figure 3** Inhibition of GSH transferase A2-2 by CHBSG. The activity of GSH transferase A2-2 towards CDNB in the presence or absence of CHBSG was measured and analysed as described in the text. **a**, Dixon plot obtained in the presence of 1 mM GSH, 1 mM CDNB and several concentrations of CHBSG; **b**, Hanes plot of data obtained with 1 mM GSH, 1 mM CDNB in the presence (■) or absence (▲) of 1.6 μM CHBSG.

tor yielding a Ki of 5.2 ± 1.1(SEM μM for GST A1-1, while CHB did not inhibit significantly in this range.

GSTs P1-1 and M1a-1a were only significantly inhibited by CHBSG at concentrations greater than 10 μM.

*Effect of GST A1-2 on CHB reaction at concentrations of clinical relevance*

The above experiments suggested that the low level of catalysis seen with 110 μM CHB was due to product inhibition. The question then arose of the catalytic effect of GSTs at the lower concentrations of CHB which may be attained with a chemotherapeutic dose (< 5 μM). Such assays, with an initial concentrationm of CHB of 2 μM, were carried out in a larger volume for accurate analysis and required larger amounts of GST. GST A1-2, the alpha class isoenzyme most readily available was chosen for study. The results (Figure 4) clearly show a decrease in remaining CHB in the presence of 52 μM GST A1-2, more evident at 35 than at 60 min, due to catalysis of GSH conjugation. More marked is the stabilisation of CHBSG which reached 74.3 ± 1.5(SEM)% of the total products at 60 min compared to 28.9 ± 0.3(SEM)% in the control. In assays stopped at 5 min 10.8% of CHB was conjugated with GSH in control compared with 14.9% in the presence of GST A1-2 (data not shown).

If the stabilisation of CHBSG by GST A1-2 is due to sequestration at the enzyme active site, the effect should be prevented by other inhibitors of activity. In Figure 5 is shown the results of inclusion of S-hexyl glutathione, an inhibitor of the 1-chloro-2,4-dinitrobenzene GSH transferase activity with Ki 3 μM (Mannervik & Danielson, 1988) on the reaction of 4 μM CHB in the presence of 36 μM GST A1-2. While 10 μM S-hexyl glutathione had no significant effect on CHB conjugation or CHBSG stabilisation by the GST, 100 μM of the inhibitor completely prevented the effects of the GST. Thus, in a physiological situation the relatively high molarity of the GST is an important factor, low levels of inhibitor being insufficient to titrate the enzyme present with significant effect.

To test whether GST A1-2 could sequester CHBSG in the presence of other cellular constituents a crude soluble macromolecule fraction including proteins and nucleic acids was prepared from human kidney. Serum albumin was removed since it stabilises CHB (Ehrsson et al., 1981). In Table I is shown the amounts of CHB and reaction products obtained in the presence and absence of the macromolecule fraction, and the effect of inclusion of 30 μM GST A1-2. In the presence of the macromolecule fraction (12 mg protein$^{-1}$ ml$^{-1}$) but without GST A1-2, only 58.8% of the drug was recovered as CHB, CHBSG, CHBSG2 and CHBSGOH. Losses are presumably due to alkylation of macromolecules. A comparison between the yield of CHBSG and (CHBSG2 plus CHBSGOH) in the presence and absence of the macromolecule fraction indicates that components of the macromolecule fraction are alkylated not only by CHB but also by CHBSG. In the presence of GST A1-2 the recovery of CHB and its GSH reaction products is increased to 80.9% and significant catalysis of GSH conjugation and stabilisation of CHBSG are observed.

**Figure 4** Effect of GST A1-2 on reaction of 2 μM CHB CHB (2 μM) was incubated at 37°C in the presence or absence of 36 μM GST A1-2 in physiological saline, pH 7.0 containing 5 mM GSH for 35 min (single assays) or 60 min (five assays) and products analysed by hplc all as described in the 'Methods'. Unshaded bars, no GST added; shaded bars, plus GST A1-2. Error bars indicate SD. Significant differences (Student t-test) are given by: *$P$ < 0.02 and **$P$ < 0.001.

**Figure 5** Effect of S-hexyl glutathione on the capacity of GST A1-2 to alter the reaction of CHB with GSH CHB (2 μM) was incubated at 37°C in physiological saline pH 7.0 containing 5 mM GSH for 60 min alone (open bars), with 36 μM GST A1-2 (shaded bars), with GST A1-2 + 10 μM S-hexyl glutathione (diagnonal bars) or with GST A1-2 + 100 μM S-hexyl glutathione (horizontal bars). Error bars indicate SD. Significant differences (Student t-test) are given by: *$P$ < 0.05; **$P$ < 0.002; ***$P$ < 0.001.

*Alkylation of guanine-N7*

In order to assess the toxicological significance of the sequestration of CHBSG, its ability to alkylate DNA was compared with that of CHB and CHBOH. The autoradiographs showed that, while CHBOH alkylated the plasmid as efficiently as CHB at 0.1 and 0.33 mM, CHBSG gave no detectable DNA alkylation under these conditions (data not shown).

### Discussion

The results confirm the findings of Ciaccio et al. (1991) that human alpha class GSTs catalyse the first GSH conjugation of CHB. However, it is also shown that an important effect, particularly at low concentrations of CHB, is the non-covalent sequestration of the primary product CHBSG. This also occurs with GSTs P1-1 and M1a-1a but the alpha class GSTs are most sensitive to CHBSG as judged by inhibition of activity. This interpretation is confirmed by the ability of S-hexyl glutathione to inhibit both catalysis of CHB conjugation and CHBSG sequestration of GST A1-2. Both effects were also seen in the presence of a mixture of potentially competing macromolecules, so they may well occur in cytosol. No evidence of catalysis of the second GSH conjugation was obtained. The human alpha class GSTs therefore differ markedly from the murine alpha GST studied by Ciaccio et al. (1990) which catalyses the GSH conjugation of both

**Table I** Effect of GST A1-2 on CHB reaction in the presence of a crude cellular macromolecule fraction

| Sample | *Recovery* % | *CHB* % | *CHBSG* % | *CHBSG2* % | *CHBSGOH* % |
|---|---|---|---|---|---|
| Buffer A control | 100 | 17.9 ± 2.4 | 28.3 ± 1.7 | 43.0 ± 2.7 | 10.5 ± 0.8 |
| Fraction M | 58.8 ± 1.1[a] | 17.0 ± 0.8 | 17.9 ± 0.1[a] | 18.8 ± 0.2[a] | 5.1 ± 0.1[a] |
| Fraction M + GST | 80.9 ± 2.1[b] | 14.8 ± 1.3[b] | 49.4 ± 2.5[b] | 12.9 ± 0.6[b] | 4.0 ± 0.8 |

A crude human kidney macromolecule fraction (M) lacking albumin and GSTs was prepared and incubated with 4 μM CHB in buffer A in the presence or absence of 30 μM GST A1-2. CHB and GSH reaction products were analysed as described in the Methods. Values are means of three determinations ± SD. [a]Significantly different from control, $P < 0.001$. [b]Significantly different from fraction M (no GST), $P > 0.001$.

CHB and CHBSG. Recently the primary sequence of the murine alpha GST subunit (mYc) was obtained by Beutler and Eaton (1992) and it is clearly more closely related to the rat liver GST subunit 10 (Meyer *et al.*, 1991*b*) and/or Yc2 (Hayes *et al.*, 1991) rather than the major rat alpha GST subunits 1a, 1b and 2. GST subunit 10 occurs mainly in foetal and neonatal rat liver (Tee *et al.*, 1992) and may also be induced in adult liver by 1,2-dithiole-3-thione (Meyer *et al.*, 1992) or ethoxyquin (Hayes *et al.*, 1991). It is possible that the alpha class GST associated with CHB-resistance in hamster cells (Lewis *et al.*, 1988) is also more closely related to this type of alpha class subunit. To date, a human equivalent of mYc (or rat subunit 10/Yc2) has not been discovered.

In their study of human GSTs, Ciaccio *et al.* (1991) obtained kinetic data on the catalysis of the first GSH conjugation of CHB. They obtained Km values for CHB of 19, 150, 220 and 830 μM for GSTs A1-1, A1-2, A2-2 and P1-1 respectively. These assays were carried out at lower, non-physiological, pH (6.5) which reduces the relative contribution of the non-catalytic reaction rate and were terminated at 2.5 min before the inhibitory effects of CHBSG were apparent. The results presented here show that at physiological pH; with concentrations of salts, GSH and GSTs approximating those in cells, and amounts of CHB and time periods similar to those pertaining to chemotherapy, the catalysis of CHB conjugation with GSH is of marginal significance compared to the non-catalytic reaction. However, the sequestration of CHBSG is an important effect.

The significance of CHBSG sequestration cannot be readily assessed without further knowledge if its toxicity. When CHB was incubated with a crude soluble macromolecule fraction lacking GSTs and albumin, reduced recoveries of CHBSG, CHBSG2 and CHBSGOH were attributable to the alkylation of macromolecules by both CHB and the monofunctional CHBSG. When GST A1-2 was added, both its catalytic and sequestration effects were observed and the apparent alkylation of macromolecules was considerably reduced. The apparent alkylation due to CHBSG is likely to be of proteins rather than nucleic acids since no alkylation of guanine in DNA was observed with this compound.

Since GS-conjugates do not enter cells by diffusion, it is not possible to examine the toxicity of CHBSG formed inside the cell from CHB simply by adding CHBSG to cells. It has long been known that the monofunctional form of CHB (4-[p-N-2-chloroethylaminobenzene]-butanoic acid) is as toxic to normal cells as CHB (Connors *et al.*, 1960), but to what extent protein alkylation contributes to toxicity is unknown.

Despite these uncertainties, it may be surmised that the function of GSTs A1/2 in cells treated with CHB will tend to be inhibited by CHBSG. The extent of inhibition will depend on the concentrations of both the GSTs and CHBSG. The concentration of CHBSG will in turn depend on the capacity of the cell to export the conjugate via a plasma membrane ATP-dependent system (Ishikawa *et al.*, 1990; Elferink *et al.*, 1991; Singhal *et al.*, 1991; Akerboom *et al.*, 1992). If CHBSG can be rapidly excreted without inactivating the transport system through alkylation, the presence of human alpha class GSTs at a concentration of 5–10 μM, as found for instance in ovarian carcinoma samples (Green *et al.*, 1990), would be expected to contribute significantly to CHB detoxication through direct catalysis of CHBSG formation. Protein alkylation by CHBSG should also be much reduced. If, however, CHBSG excretion is slow, CHBSG will rapidly accumulate and inhibit not only the catalytic detoxication of CHB but perhaps also other protective functions of alpha class GSTs such as the inhibition of lipid peroxidation via their selenium-independent GSH peroxidase activity (Tan *et al.*, 1984). In this case the GST should contribute little or nothing to protection against CHB toxicity. Variations in the relative concentrations of GSTs and CHBSG may thus explain the disparity between the results of Leyland-Jones *et al.* (1991) who found no CHB-protective effect of transfected human GST A1-1 in MCF-7 cells and those of for example Puchalski and Fahl (1990), who obtained positive results for rat alpha GST using transfection of mouse (1.2-fold-resistant) and monkey cells (1.4-fold-resistant).

In order to achieve sensitisation to CHB of resistant cells, GST inhibitors such as ethacrynic acid have been used (Tew *et al.*, 1988; Hansson *et al.*, 1991). Moreover, ethacrynic acid was recently used to inhibit GSTs in a phase I study (O'Dwyer *et al.*, 1991). The basis for such use was the low Ki value for human GSTs with ethacrynic acid and its GS-conjugate in the range of 0.1–6 μM (Ploeman *et al*, 1990). However, it is clear from the data presented here that a Ki of about 1 μM is of little significance when the cellular concentration of GST is for example, 10 μM as found in ovarian carcinoma samples (Green *et al.*, 1990). Thus 10 μM S-hexyl glutathione had no detectable effect on GST A1-2 when the latter was in molar excess (Figure 5). The sensitisation to CHB due to ethacrynic acid may result not only from direct inhibition of activity, but also from competition between the GS-conjugates of ethacrynic acid and CHB for excretion. The studies presented in this paper emphasise that to inhibit GSTs *in vivo* it is important to consider both the relatively high molar concentration of these enzymes and cellular capacity to excrete GSH conjugates.

The authors are grateful to B. Coles for helpful discussions. This work was supported by the Cancer Research Campaign.

### References

AKERBOOM, T.P.M., BARTOSZ, G. & SIES, H. (1992). Low and high Km transport of dinitrophenyl glutathione in inside-out vesicles from human erythrocytes. *Biochim. Biophys. Acta*, **1103**, 115–119.

BANK, B.B., KANGANIS, D., LIEBES, L.F. & SILBER, R. (1989). Chlorambucil pharmacokinetics and DNA binding in chronic lymphocytic leukemia lymphocytes. *Cancer Res.*, **49**, 554.

BEUTLER, M.T. & EATON, D.L. (1992). Complementary cDNA cloning, mRNA expression, and induction of alpha class glutathione S-transferase in mouse tissues. *Cancer Res.*, **52**, 314.

BRADFORD, M.M. (1976). A rapid and sensitive method for the quantitation of microgram quantities of protein utilizing the principle of protein-dye binding. *Anal. Biochem.*, **72**, 248.

CIACCIO, P.J., TEW, K.D. & LACRETA, F.P. (1990). The spontaneous and GSH S-transferase-mediated reaction of chlorambucil with glutathione. *Cancer Commun.*, **2**, 279.

CIACCIO, P.J., TEW, K.D. & LACRETA, F.P. (1991). Enzymatic conjugation of chlorambucil with glutathione by human glutathione S-transferases and inhibition by ethacrynic acid. *Biochem. Pharmacol.*, **42**, 1504.

CMARIK, J.L., INSKEEP, P.B., MEREDITH, M.J. & 3 others (1990). Selectivity for rat and human glutathione S-transferases in activation of ethylene dibromide by glutathione conjugation and DNA binding and induction of unscheduled DNA synthesis in human hepatocytes. *Cancer Res.*, **50**, 2747.

CONNORS, T.A., ROSS, W.C.J. & WILSON, J.G. (1960). Aryl-2-halogenoalkylamines. Part XIX. Some N,N-di-2-chloroethyl-amino-phenyl and phenylalkyl-hydantoins and related amino acids. *J. Chem. Soc.*, **604**.

EHRSSON, H., LÖNROTH, U., WALLIN, I., EHRNEBO, M. & NILSSON, S.O. (1981). Degradation of chlorambucil in aqueous solution-influence of human albumin binding. *J. Pharmacol.*, **33**, 313.

GREEN, J.A., HARRIS, J., BRITTEN, R. & 3 others (1990). Glutathione S-transferase (GST) isoenzyme expression in normal and malignant human ovarian biopsies. *Proc. Amer. Assoc. Cancer Res.*, **31**, 7.

HABIG, W.H., PABST, M.J. & JAKOBY, W.B. (1974). Glutathione S-transferases the first step in mercapturic acid formation. *J. Biol. Chem.*, **249**, 7130.

HANSSON, J., BERHANE, K., CASTRO, B.M. & 3 others (1991). Sensitization of human melanoma cells to the cytotoxic effect of melphalan by the glutathione transferase inhibitor ethacrynic acid. *Cancer Res.*, **51**, 94.

HARTLEY, J.A., GIBSON, N.W., KOHN, K.W. & MATTESS, W.B. (1986). DNA sequence selectivity of guanine-N7 alkylation by three antitumour chloroethylating agents. *Cancer Res.*, **46**, 1943.

HAYES, J.D., JUDAH, D.J., MCLENNAN, L.I. & 3 others (1991). Ethoxyquin-induced resistance to aflatoxin B1 in the rat is associated with the expression of a novel alpha class glutathione S-transferase subunit Yc2, which possesses high catalytic activity for aflatoxin B1-8,9-oxide. *Biochem.*, **279**, 385.

HUSSEY, A.J., STOCKMAN, P.K., BECKETT, G.J. & HAYES, J.D. (1986). Variations in the glutathione S-transferase subunits expressed in human livers. *Biochim. Biophys. Acta*, **874**, 1.

ISHIKAWA, T., MULLER, M., KLUNEMANN, C., SCHAUB, T. & KEPPLER, D. (1990). ATP-dependent primary active transport of cysteinyl leukotrienes across liver canalicular membrane. *J. Biol. Chem.*, **265**, 19279.

JOHNSTON, J.B., ISRAELS, L.G., GOLDENBERG, G.J. & 4 others (1990). Glutathione S-transferase activity, sulfhydryl group and glutathione levels, and DNA cross-linking activity with chlorambucil in chronic lymphocytic leukemia. *J. Natl Cancer Inst.*, **82**, 776.

KETTERER, B., CARNE, T. & TIPPING, E. (1978). Ligandin and protein A: intracellular binding proteins. In *Transport by Proteins*, Blauer, G. & Sund, H. (eds) pp. 78–94. Walter de Gruyter: Berlin.

LEWIS, A.D., HICKSON, I.D., ROBSON, C.N. & 7 others (1988). Amplificatiion and increased expression of alpha class glutathione S-transferase-encoding genes associated with resistance to nitrogen mustards. *Proc. Natl Acad. Sci. USA*, **85**, 8511.

LEYLAND-JONES, B.R., TOWNSEND, A.J., TU, C.-P.D., COWAN, K.H. & GOLDSMITH, M.E. (1991). Antineoplastic drug sensitivity of human MCF-7 cancer cells stably transfected with a human α class glutathione S-transferase gene. *Cancer Res.*, **51**, 587.

LISTOWSKY, I., ABRAMOWITZ, M., HOMMA, H. & NIITSU, Y. (1988). Intracellular binding and transport of hormones and xenobiotics by glutathione S-transferases. *Drug Metab. Rev.*, **19**, 305–318.

MANNERVIK, B. & DANIELSON, U.H. (1988). Glutathione transferases – structure and catalytic activity. *CRC Crit. Rev. Biochem.*, **23**, 281.

MANNERVIK, B., AWASTHI, Y.C., BOARD, P.G. & 11 others (1992). Nomenclature for human glutathione transferases. *Biochem. J.*, **292**, 305.

MEYER, D.J., LALOR, E., COLES, B. & 4 others (1989). Single step purification and h.p.l.c. analysis of GSH transferase 8-8 in rat tissues. *Biochem. J.*, **260**, 785.

MEYER, D.J., COLES, B., PEMBLE, S.E. & 3 others (1991*a*). Theta, a new class of glutathione transferases purified from rat and man. *Biochem. J.*, **274**, 409.

MEYER, D.J., GILMORE, K.S., COLES, B. & 3 others (1991*b*). Structural distinction of rat glutathione transferase subunit 10. *Biochem. J.*, **274**, 619.

MEYER, D.J., COLES, B., HARRIS, J. & 6 others (1992). Induction of rat liver GSH transferases by 1,2-dithiole-3-thione illustrates both anticarcinogenic and tumour-promoting properties. In *Antimutagenesis and Anticarcinogenesis Mechanisms, III*, Bronzetti, G., Hayatsu, H., DeFlora, S., Waters, M.D. & Shankel, D.M. (eds) Plenum Press: New York (in press).

NAKAGAWA, K., SAIJO, N., TSUCHIDA, S. & 7 others (1990). Glutathione-S-transferase π as a determinant of drug resistance in transfectant cell lines. *J. Biol. Chem.*, **265**, 4296.

O'DWYER, P.J., LACRETA, F., NASH, S. & 8 others (1991). Phase I study of thiotepa in combinations with the glutathione transferase inhibitor ethacrynic acid. *Cancer Res.*, **51**, 6059.

OUDE ELFERINK, P.J., OTTENHOF, R., RADOMINSKA, A. & 3 others (1991). Inhibition of glutathione-conjugate secretion from isolated hepatocytes by dipolar bile acids and other organic anions. *Biochem. J.*, **274**, 281.

PLOEMAN, J.H.T.M., VAN OMMEN, B. & VAN BLADEREN, P.J. (1990). Inhibition of rat and human glutathione S-transferase isoenzymes by ethacrynic acid and its glutathione conjugate. *Biochem. Pharmacol.*, **40**, 1631.

PUCHALSKI, R.B. & FAHL, W.E. (1990). Expression of recombinant glutathione S-transferase π, Ya or $Yb_1$ confers resistance to alkylating agents. *Proc. Natl Acad. Sci. USA*, **87**, 2443.

SINGHAL, S.S., SHARMA, R., GUPTA, S. & 5 others (1991). The anionic conjugates of bilirubin and bile acids stimulate ATP hydrolysis by S-(dinitrophenyl)glutathione ATPase of human erythrocyte. *FEBS Lett.*, **281**, 255.

TEE, L.B.G., GILMORE, K.S., MEYER, D.J. & 3 others (1992). Expression of glutathione S-transferase during liver development. *Biochem. J.*, **282**, 209.

TAN, K.H., MEYER, D.J., BELIN, J. & KETTERER, B. (1984). Inhibition of microsomal lipid peroxidation by glutathione and glutathione transferases B and AA. *Biochem. J.*, **220**, 243–252.

TEW, K.D., BOMBER, A.M. & HOFFMAN, S.J. (1988). Ethacrynic acid and piriprost as enhancers of toxicity in drug resistant and sensitive cell lines. *Cancer Res.*, **48**, 3622.

VANDER-JAGT, D.L., HUNSAKER, L.A., GARCIA, B. & ROYER, R.E. (1985). Isolation and characterization of the multiple glutathione S-transferases from human liver. *J. Biol. Chem.*, **260**, 11603.